# A Novel Modification of the Melody Valve in a Short Right Ventricle-Pulmonary Artery Conduit: A Case Report

**DOI:** 10.1016/j.jscai.2024.102505

**Published:** 2025-01-18

**Authors:** Marjan Hesari, Nissma Bencheikh, Danica Peterson, Justin R. Ryan, Kamel Shibbani, Clinton Fulk, Howaida El-Said

**Affiliations:** a Department of Pediatrics, University of California San Diego, La Jolla, California; b Division of Cardiology, Rady Children’s Hospital-San Diego, San Diego, California; c School of Medicine, University of California San Diego, La Jolla, California; d Helen and Will Webster Foundation 3D Innovations Lab, Rady Children’s Hospital, San Diego, California; e Department of Neurological Surgery, UC San Diego Health, La Jolla, California

**Keywords:** Melody valve, pulmonary regurgitation, simultaneous stenting, valve modification

## Abstract

Pulmonary insufficiency often follows the surgical repair of tetralogy of Fallot, leading to adverse outcomes. Young patients with short right ventricle-pulmonary artery conduits are at risk of pulmonary artery branch occlusion when a traditional Melody valve (Medtronic) is used. We report a novel case of a folded Melody valve implanted with a simultaneous stent in a pediatric patient to address challenges posed by a short right ventricle-pulmonary artery conduit.

## Introduction

The Melody valve (Medtronic), a stented bovine jugular venous graft, is frequently used for transcatheter pulmonary valve replacement (TPVR) in the pediatric population. US Food and Drug Administration-approved for right ventricle to pulmonary artery (RV-PA) conduits, the valve’s length can challenge use in young patients with small right ventricular outflow tracts (RVOTs).^1^ Typical challenges include occlusion risk to branch pulmonary arteries and difficulty achieving ideal deployment. Stent fractures (SFs) and endocarditis remain significant complications in TPVR.^2^ Simultaneous prestenting is sometimes adopted to improve stability and reduce complications.^3^ Here, we present a case that employed a modified Melody valve with simultaneous stenting within a short RV-PA conduit to alleviate pulmonary insufficiency (PI) in a young tetralogy of Fallot (ToF) patient.

## Case report

### Patient background

The patient was a 7-year-old boy with DiGeorge Syndrome, ToF, pulmonary atresia, a single major aortopulmonary collateral artery, and a right aortic arch. He had a history of ventricular septal defect patch closure, subpulmonary muscle bundle resection, major aortopulmonary collateral artery unifocalization, and placement of a 17-mm RV-PA conduit. Progressive right ventricular dilation due to symptomatic severe PI warranted intervention.

### Procedure

**Figure 1:**
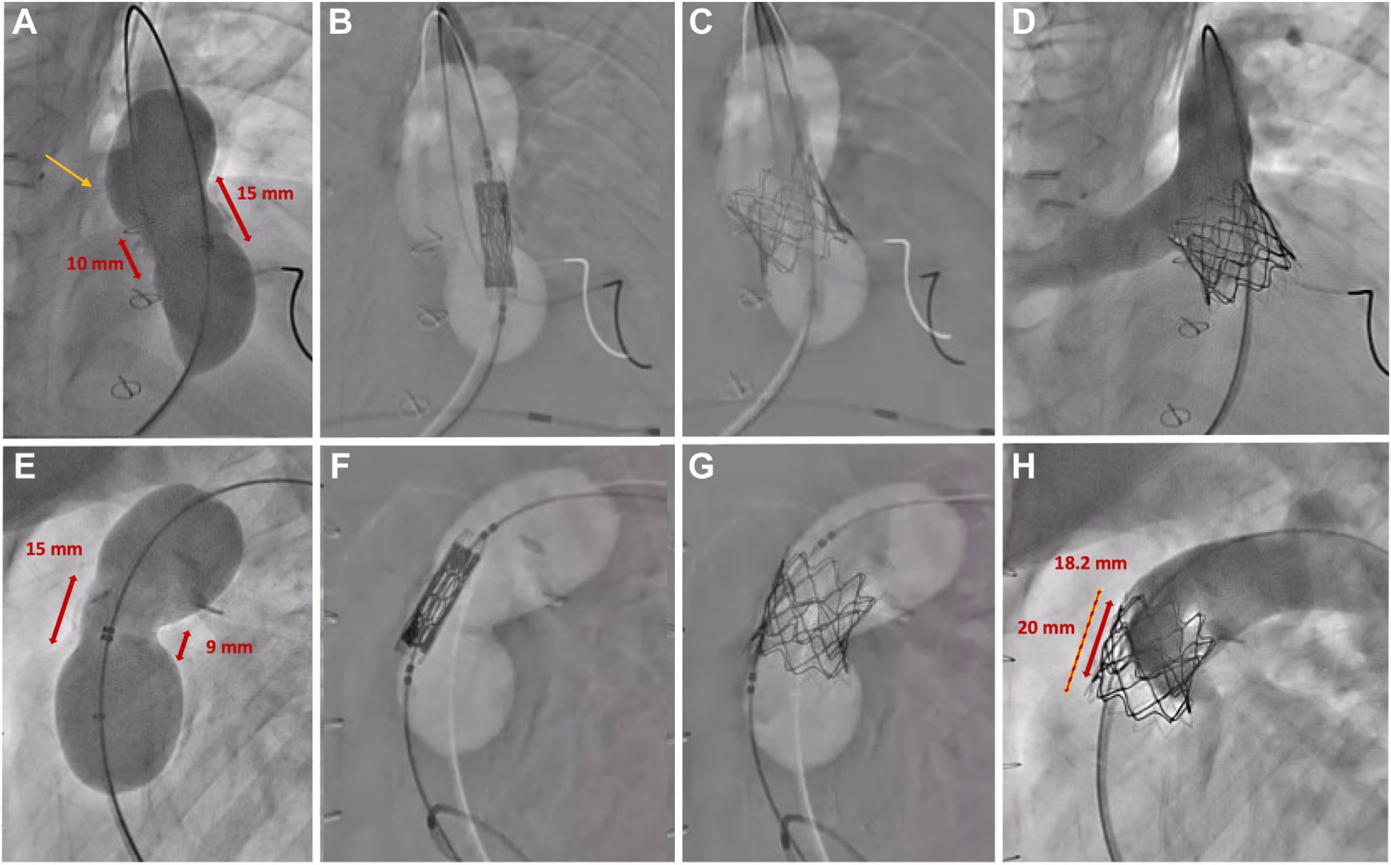
The upper panels show anteroposterior views, while the lower panels depict lateral views of angiograms taken during the procedure. (**A, E**) Balloon sizing angiograms reveal a very short and asymmetric landing zone, measuring approximately 15 mm anteriorly and leftward and 9 to 10 mm posteriorly and rightward. A bulge in the sizing balloon is observed (yellow arrow), indicating the location of the right pulmonary artery (RPA). (**B, F**) An overlay image of the sizing balloon, a shortened Melody valve, and a Palmaz stent mounted on a 20-mm Melody balloon Ensemble placed over the Lunderquist wire. (**C, G**) Angiograms showing the shortened Melody valve and Palmaz stent fully expanded in the target position, with the sizing balloon overlay still visible. (**D, H**) A final angiogram performed with a Multitrack catheter in the main pulmonary artery confirms the valve’s stable positioning with no regurgitation and a widely patent RPA. The shortened Melody valve’s final diameter is 18.2 mm, and the Palmaz stent’s diameter is 20 mm.

**Figure 2:**
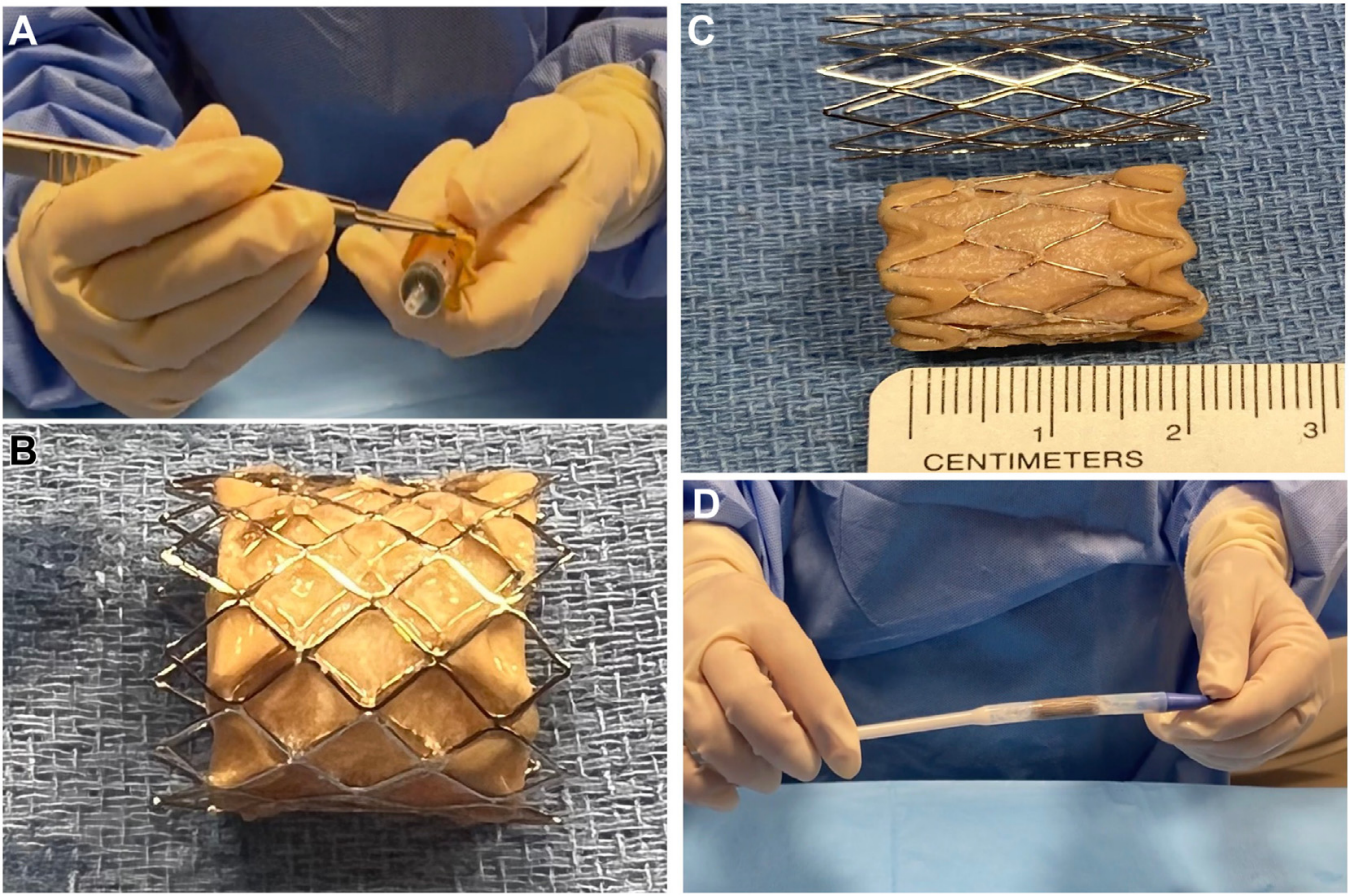
(**A**) Shortening Melody valve before implantation. (**B, C**) Modified Melody valve measuring approximately 20 mm. (**D**) Modified Melody valve with overlying Palmaz stent, loaded into the Ensemble delivery system.

Due to the short asymmetric conduit (15 mm on one side and 10 mm on the other), a traditional percutaneous valve replacement risked the occlusion of the right pulmonary artery (RPA) (Figure 1). Therefore, a modified “shortened” Melody valve, with simultaneous stenting, was performed (Figure 2).

### Valve preparation

**Figure 3:**
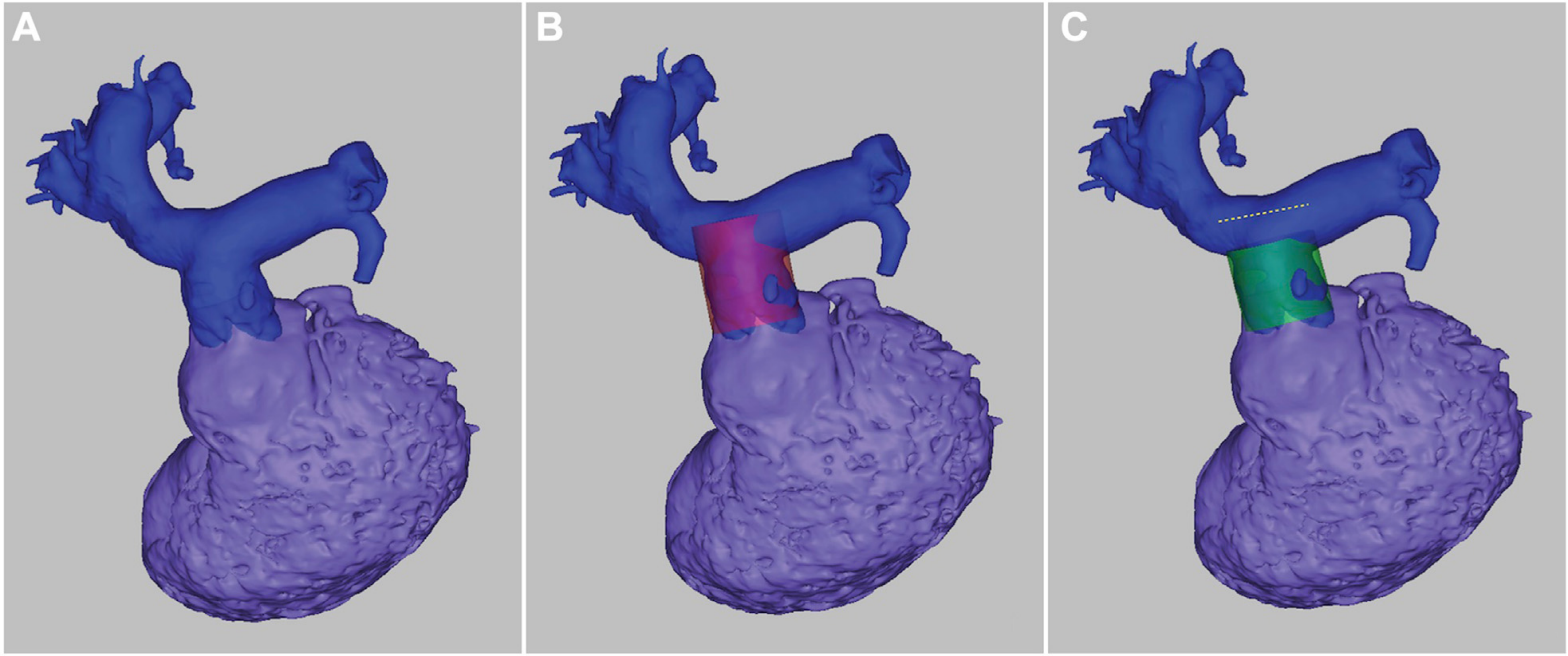
3D reconstruction of the right ventricular outflow tract (RVOT), main pulmonary artery (MPA), and branch pulmonary arteries (PAs), demonstrating a simulation of Melody valve placement with and without shortening. (**A**) Baseline 3D reconstruction of the RVOT, MPA, and branch PAs. (**B**) Simulation of Melody valve placement without shortening (purple). This configuration shows that the valve encroaches on the right pulmonary artery. (**C**) Simulation of Melody valve placement with shortening (green). The shortened valve avoids obstruction of the right pulmonary artery, demonstrating improved spatial accommodation.

A 3D reconstruction from computed tomography imaging aided procedural planning (Figure 3). Computed tomography angiography suggested a standard Melody valve would obstruct the RPA. Shortening the valve from its original size of 28 to 30 mm to 20 mm would allow safe placement. The shortening was performed by folding the valve’s top and bottom struts (Video 1). A 20-mm Melody Ensemble balloon was used, with a Palmaz 3110 stent (Cordis) crimped over the shortened Melody valve to ensure secure placement (Figure 2).

### Implantation

A Lunderquist wire (Cook Medical) placed in the left pulmonary artery facilitated the deployment of the shortened Melody valve–Palmaz stent unit within the RVOT, with pacing used to enhance stability. Postdeployment angiography confirmed an optimal position with no residual PI and patent RPA. On fluoroscopy post deployment, the Melody valve itself measured 18.2 mm and the Palmaz stent 20 mm (Figure 1).

## Discussion

PI remains a common sequela of surgical repair of ToF, increasing the risk of complications.^4^ Pulmonary valve replacement, often percutaneous, has emerged as the preferred procedure to address symptomatic cases.^5^ The Melody valve was selected in this case due to its size, ability to withstand modification, and proven efficacy in mitigating residual PI.^6^ Folded Melody valves in the mitral and pulmonary positions have been shown to have results comparable to nonmodified Melody valves.^7^

Jalal et al^7^ reported 49 cases in which the Melody valve was shortened and placed in the pulmonary position with a reintervention rate of 2.1%. The primary reasons for reintervention included endocarditis and folded Melody SF.^7^ Based on clinical trials, a significant complication of TPVR using the Melody valve is SFs, which are estimated to affect 25% of patients. This is still the leading cause of RVOT reinterventions.^8^ Moreover, a meta-analysis revealed that prestenting with the Melody valve provides better protection against SFs; however, the optimal number of prestents remains unknown.^9^ Prestent provides a stable platform for the valve, which has been shown to improve procedural success.^10^ However, staged prestenting can result in the dislodgement of the prestent while advancing the Melody valve.^3^ To enhance implantation stability, prevent stent dislodgement, and reduce the risk of SF—particularly in our case, with a very short landing zone—we employed the simultaneous stenting technique along with Melody valve modification and shortening.

Our report demonstrates that the shortened Melody valve can be implanted simultaneously with a landing zone stent on the Melody Ensemble balloon to mitigate the risk of migration of the stent, shorten the procedure, and reduce total radiation exposure. The concurrent use of a stent in this modified procedure also allowed for greater precision and stability of the valve placement in short RV-PA conduits.

## Conclusion

This case demonstrates successful use of a shortened Melody valve with simultaneous stenting in a young patient with a short RV-PA conduit. This technique is a promising option for similar complex cases.

## Supplementary material

Video 1 This video demonstrates the Melody valve preparation process. The Melody valve is first dilated and positioned over a 5-mL syringe to aid in crimping the upper and lower stent struts. Once the struts are folded, the shortened Melody valve is removed from the syringe and positioned on the 20-mm Ensemble, ensuring correct orientation. The modified Edwards crimper is then used to secure the Melody valve onto the Ensemble. Next, a 3010 Palmaz Stent is dilated and placed over the shortened Melody valve, with the Edwards crimper again used to secure the stent onto the Melody valve. The Ensemble’s sheath is then carefully advanced over the Palmaz and shortened Melody valve combination, ensuring central positioning on the balloon. Finally, the sheath covers both the stent and the shortened Melody valve, and the Ensemble is curved to facilitate delivery over the Lunderquist wire.
